# Emergence of intercolonial variation in termite shelter tube patterns and prediction of its underlying mechanism

**DOI:** 10.1098/rsos.150360

**Published:** 2015-11-04

**Authors:** Nobuaki Mizumoto, Kazuya Kobayashi, Kenji Matsuura

**Affiliations:** Laboratory of Insect Ecology, Graduate School of Agriculture, Kyoto University, Kyoto 606-8502, Japan

**Keywords:** collective behaviour, self-organization, colony variation, nest building, stigmergy, social insects

## Abstract

Building behaviours occur in various organisms from bacteria to humans. Social insects build various structures such as large nests and underground galleries, achieved by self-organization. Structures built by social insects have recently been demonstrated to vary widely in size and shape within a species, even under the same environmental conditions. However, little is known about how intraspecific variation in structures emerges from collective behaviours. Here we show that the colony variation of structures can be generated by simply changing two behavioural parameters of group members, even with the same building algorithm. Our laboratory experiment of termite shelter tube construction demonstrated clear intercolonial variation, and a two-dimensional lattice model showed that it can be attributed to the extent of positive feedback and the number of individuals engaged in building. This study contributes to explaining the great diversity of structures emerging from collective building in social insects.

## 1. Introduction

Many animals have adapted to build structures that modify their environments [1]. In social insects, structures have various functions such as protection against predators or harsh environments, ventilation, thermoregulation and space for food storage or fungus cultivation [1–4]. Construction is achieved by self-organization, whereby colony-level structures emerge from local interactions among members that elicit positive and negative feedback responses [5,6]. In this building system, stigmergy plays an important role [7–9], with local interactions among individuals occurring indirectly through environment modification. Many studies have shown that this building rule can produce the complex structures observed in various social insect species [5,10,11] and the interspecific variation observed in nature [12]. A recent study demonstrated that structure differences occur not only among species, but also among colonies within a species [13]. However, little is known about how intercolonial variation emerges from collective building with the same behaviours.

Several studies have revealed two main types of factors that create intercolonial variation in self-organized construction: environmental factors and colony size. Environmental factors, such as temperature, materials and flow of wind, influence individual worker responses and structure morphology [11,14–16]. Colony size also affects the size and shape of structures by regulating construction activity [17,18]. However, we have recently shown that termite workers construct colony-specific structures even under identical environments and group sizes [13], which cannot be explained by known factors. In this paper, we focused on mechanisms producing colony-specific structures to further the understanding of intraspecific structure diversity.

Termite construction is a classic example of stigmergic building, whereby interactions among individuals are often mediated by the cement pheromone [11,19]. This pheromone is added when building material is attached, attracting workers to continue building in the same location. An accumulation of material induces an accumulation of the pheromone, which attracts even more termite workers. This positive feedback results in the construction of various structures such as pillars, walls and shelter tubes [11,12]. The Japanese subterranean termite *Reticulitermes speratus* is classified as a multiple-piece nester, wherein the nests of a single colony are interconnected by below-ground tunnels and above-ground shelter tubes [20,21]. Shelter tubes are made of wood, soil and termite excretions, which provide shelter and protection from predators and the external environment. This species shows colony-specificity in shelter tube construction, that is, subdivided groups of workers from the same colony show similar patterns of shelter tube construction, whereas groups derived from different colonies show distinctly different construction patterns. For example, some groups form no shelter tubes but simply cover the bottom of the container with mats, other groups construct many shelter tubes and still others construct fewer but longer shelter tubes [13]. This provides an excellent model system for studying structure variation among colonies.

To identify intercolonial variation in construction, we quantified the structural character of shelter tubes built by groups of individuals taken and divided from several field colonies. Then, to examine factors influencing collective building and structure, we developed a two-dimensional lattice construction model, where we changed the characteristics of individual workers. We prepared two building conditions (groups of 50 or 400 workers) to develop a robust model which can be applied to broad conditions. By comparing the observed structures constructed by termites with simulation results, we explored a possible mechanism which can explain how intraspecific variation in structures emerges from collective behaviours.

## 2. Methods and results

### 2.1 Empirical experiments

We collected five colonies of *R. speratus* during August–October 2013, from pine–oak forests at Yoshida (colony codes: A–D) in the northern suburbs of Kyoto, Japan, and at Zeze (colony code: E) in southern Shiga, Japan. All colonies were brought back to the laboratory with colonized wood, and termites were collected and used for experiments within 3 days. We used third-stage or larger workers to construct shelter tubes because larvae and small workers are unable to build them [22]. Groups of 50 and 400 workers were randomly selected from each colony and placed on a block of mixed sawdust (36 mm diameter×15 mm height) at the centre of a long plastic container (221×141×37 mm). The sawdust block was prepared from brown rotten pinewood and cellulose powder (Nacalai Tesque, Kyoto, Japan) and mixed at a ratio of 5 : 1 by volume as construction material and food. We made five replicates of 400 workers and five replicates of 50 workers from each of five colonies. Thus, we used 2250 workers from each colony, in total. The plastic containers were maintained at 25°C under a 16 L : 8 D photoperiod for 30 days. The termites constructed shelter tubes from the block and/or covered the bottom of their container with mats consisting of particles of mixed sawdust and faeces (electronic supplementary material, figure S1). Two types of shelter tubes were constructed: an edging shelter tube and a non-edging shelter tube. The former type was constructed along the edge of container, and the latter was laid in the middle of the bottom of the plate (electronic supplementary material, figure S1). After 30 days, we photographed each plastic container with a digital camera (D300, Nikon, Tokyo, Japan) to record resulting structures. We then disassembled the nests and counted the number of surviving individuals. Dead individuals were buried with mixed sawdust and faeces in the containers (electronic supplementary material, figure S2). Because a large amount of the materials was used to bury the corpses in the groups with low survival rate, shelter tube construction can be affected by high mortality. To remove the effect of undertaking behaviour, we excluded data of groups whose survival rates were less than or equal to 0.5 from following analysis (electronic supplementary material, table S1).

Termites constructed two main types of structures: shelter tubes and mats. After construction, we analysed the morphology of these structures. We adjusted photographs so that 1 pixel represented approximately 1 mm (220×140 pixels), converted them to black-and-white so that brown areas (nest construction) became white and backgrounds became black. To measure the perimeters of the white areas, we eliminated small pieces of sawdust, faeces or individuals that were less than 50 pixels and small holes in structures that were less than 10 pixels (electronic supplementary material, figure S3). To analyse the structures, we measured the perimeters (mm) and the areas (mm^2^) of nest constructions. Perimeters were separately measured along edges (3 mm from edge of the container) and away from edges (electronic supplementary material, figure S3). To examine intercolonial variation in structures, we tested differences in perimeters and areas using one-way ANOVA for each group size, respectively. All analyses were performed using the ‘EBImage’, ‘biOps’ and ‘car’ packages implemented in R v. 3.1.2 (R Development Core Team, Vienna, Austria) and Adobe Photoshop v. 11.0.2 (Adobe Systems, Inc., San Jose, CA, USA).

**Figure 1.**
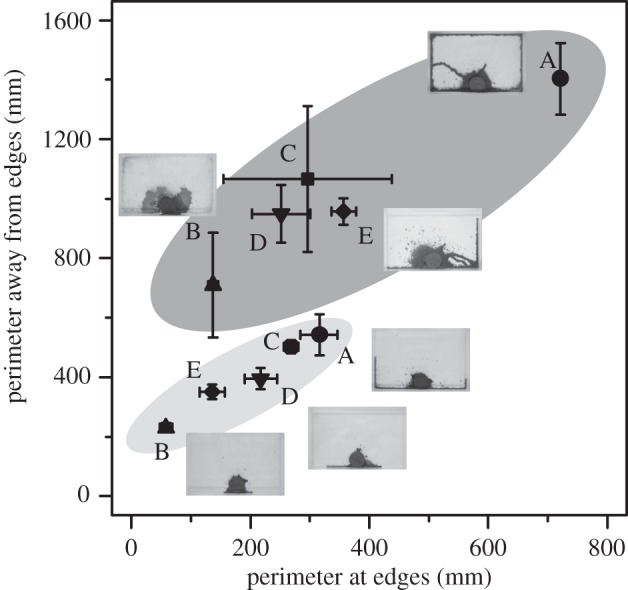
Colony variation in termite shelter tube construction. The structures are represented by perimeters along edges and away from edges. Plots in the dark grey area are patterns created by groups of 400 workers, and in the light grey area are by groups of 50 workers. Termites constructed distinctly different patterns among colonies. The data of groups with survival rates of less than or equal to 0.5 were excluded. Bars represent means±s.e.

Of the analysed 21 groups of 400 workers, seven groups from three colonies (colonies A, C and E) constructed shelter tubes, six from two colonies (colonies B and D) only covered the container surface with mats and nine from three colonies (colonies C, D and E) showed intermediate patterns. We found significant differences among colonies in the perimeters (ANOVA: *F*_4,16_=4.911, *p*=0.009), but no differences in the areas (ANOVA: *F*_4,16_=0.976, *p*=0.448) of nest construction. The differences in the perimeters were driven by differences in perimeters along the edge (figure 1; ANOVA, along edge: *F*_4,16_=9.259, *p*<0.001; away from edge: *F*_4,16_=2.281, *p*=0.106).

On the other hand, of the analysed 24 groups of 50 workers, 22 groups from all colonies constructed shelter tubes and two from two colonies only covered the container surface with mats (colonies B and E). There were significant differences among colonies in both of the perimeters (ANOVA: *F*_4,18_=18.064, *p*<0.001) and the areas (ANOVA: *F*_4,18_=15.017, *p*<0.001) of nest construction. We found significant differences in the perimeters along the edge and away from the edge (figure 1; ANOVA, along edge: *F*_4,18_=23.896, *p*<0.001; away from edge: *F*_4,18_=12.313, *p*<0.001).

### 2.2 Simulations

To understand what caused the observed intercolonial variation in structure even under the same environmental conditions and colony size, we examined factors influencing collective building. In shelter tube construction, termite workers carry pieces of wood to the end of the shelter tube and attach them one after another (see the electronic supplementary material, video S1V). Both mats and shelter tubes are constructed via the same behaviour, so stigmergic interaction should occur through the cement pheromone. Using our experimental conditions and observations of worker behaviour, we developed a two-dimensional lattice model in which multiple agents (i.e. workers) expand their structures by filling vacant lattices.

The simulations were performed using lattices of square cells (220×140), so a lattice is roughly equivalent to a 1×1 mm square. Each simulation starts with an initial circular nest area at the centre of the long side of a container, corresponding to the experimental set-up (radius=18, area=1020). As this nest area is the equivalent of the sawdust block in the experiments, this is assumed to have volume and can provide the materials of construction to the agents. At the beginning of each simulation, 50 or 400 agents are set on the nest and activated at rate of *α*. Here, we defined the activities of agents as engagement in construction, so only active workers engage in construction and the other inactive workers do nothing. For simplification, we did not consider the structures created by digging inside the sawdust block. Therefore, all agents are initially in the nest area. During a time step, each activated agent chooses one vacant lattice *i* that is adjacent to the existing structure at probability of *P*_*i*_. The probability of choosing the vacant lattice *i* (*P*_*i*_) is determined by attraction without pheromone and the quantity of cement pheromone. The value of cement pheromone for the vacant lattice *i* is defined as the largest cement pheromone value of the constructed lattices neighbouring the lattice *i* (electronic supplementary material, figure S4). All lattices have no pheromones at the beginning of each simulation. After choosing a lattice, the agent fills the lattice (puts a piece of material in the lattice) and adds *q* amount of pheromone to the lattice. At the end of each time step, the quantity of pheromone is reduced because of evaporation at a rate of *r*. We used 0.3 for *q* and 0.7 for *r* to re-create the observed patterns. We presented a sensitivity analysis of the model, testing the effects of the *q* and *r* values on the results (electronic supplementary material, appendix A). Each simulation lasts until 5700 lattices are filled for 400 agents and 1350 lattices for 50 agents, based on the average area constructed in the experiment at 30 days (mean±s.e., 400 workers: 5701.6±224.92 mm^2^; 50 workers: 1368.21±106.85 mm^2^). The flowchart of simulations is shown in the electronic supplementary material figure S5.

According to observed construction behaviour, the choice rule of lattice construction is set as follows. A candidate lattice must be a neighbour to an already-constructed lattice or to a nest. To mimic the stigmergic interaction in natural shelter tube construction, the choice probability of each lattice *i* is computed as

$$P_{i}=\frac{A_{i}}{\sum_{j}^{S}A_{j}},$$

where *A*_*i*_ is attraction to lattice *i* and *S* is the number of all open lattices that are adjacent to the existing structure. The attraction of each lattice can be given by

$$A_{i}=v_{i}{\left(c_{i}+k_{i}-bd_{i}\right)}^{x},$$

where *c*_*i*_ is the quantity of cement pheromone in the lattice and *k*_*i*_ represents the lattice preference irrespective of pheromone quantity. We chose *k* values so that the lattices around the edges of containers have twice as high values as the other lattices, because termites can construct edging shelter tubes with half the amount of material compared with non-edging shelter tubes. We set *k* values at 2 for four or five lattices from the edge of the container for 50 or 400 workers, respectively, and 1 for the other lattices, based on the average width of edging shelter tubes (mean, 400 workers: 4.86 mm; 50 workers: 4.28 mm). We presented how the value of *k* around edges of the container affects the structure in an appendix (electronic supplementary material, appendix A). *d*_*i*_ is the distance of the shortest route from the nest to the lattice *i*, *b* is a constant of 1/360, which is *k*_*i*_−*bd*_*i*_=0 when all edges of a container are constructed, and *x* is the sensitivity to the cement pheromone with the nonlinear choice effect, in which a high value indicates a high probability that the agent will choose the lattice. In our simulation, all individuals in a certain group have the same *x* value. Finally, *v*_*i*_ is the effect of construction direction of the lattice on the attraction. We included this effect because we observed that shelter tubes were constructed in a direction away from the nest. This is plausible considering that shelter tubes are formed for foraging outside of nests. To include this effect, we attached 0–1 to all directions of construction simply by using a cosine curve. The parameter of construction direction *v*_*i*_ is set as

$$v_{i}=\frac{1}{2}+\frac{1}{2}\cos\theta ,$$

where *θ* is the direction of construction, that is, the angle between the direction to the last-constructed lattice from the first-constructed lattice and the direction to the candidate lattice *i* from the last-constructed lattice (see the electronic supplementary material, figure S6). Thus, if the candidate lattice *i* is in the opposite direction relative to the first-constructed lattice as opposed to the last-constructed neighbour lattice, *v*_*i*_ is 1. If lattice *i* is in the same direction relative to the first-constructed lattice, *v*_*i*_ is 0. The first-constructed lattice is determined when a lattice neighbouring the nest or at the edge of the container is constructed. If lattice *i* has two possible first-constructed lattices, their centre is set as the beginning point of construction. The direction towards the non-edge area from the edge area was set as 0.0007274 based on experimental data. If multiple values of *v*_*i*_ at the lattice *i* can be defined, we set the sum of them as the value of *v*_*i*_ to calculate *A*_*i*_. As some other rules generate 0–1 responding to the direction, we used another rule of construction directions and this did not affect the results qualitatively (electronic supplementary material, appendix A).

Based on actual termite behaviour, we incorporated two rules into our simulation: (i) collision rule of shelter tubes; in observations, when shelter tubes collided, construction ended. Thus, we defined lattices that differed by less than 10 in *d*_*i*_ as belonging to different shelter tubes. When different shelter tubes were connected by more than or equal to 8 lattices, shelter tube construction ended by setting *A*_*i*_ as 0 in 8×8 lattices around the last constructed lattice; (ii) effect of crowdedness within a shelter tube; many workers cannot pass through the same shelter tube simultaneously. In our simulation, we set crowdedness as the average value of crowdedness in constructed lattices neighbouring the lattice, and lattices with high crowdedness (more than or equal to 0.9) were excluded. Crowdedness increases with construction in the same way as pheromone accumulation. At the end of each time step, it is reset because agents return to the nest area after construction to get a supply of materials. In our simulations, this effect of crowdedness is the only assumption of physical interactions between workers.

In our study, we modified two parameters: (i) the proportion *α* of the active workers engaging in construction in a group, and (ii) sensitivity *x* of workers to the cement pheromone, which can vary among colonies but not among individuals. Here, we used 25 sets of two parameters to describe variation in worker characteristics. We used five values of *α* ranging from 0.05 to 0.25 at an interval of 0.05 and five values of *x* ranging from 1 to 3 at an interval of 0.5. For each parameter set, we ran 100 simulations and recorded the patterns of every 1000 constructed lattices. Simulation results were analysed in the same way as the shelter tube experiment. We performed all simulations using Visual Studio C++ 2012. We provide the code of simulations and the typical growth patterns of structures in the electronic supplementary material, appendix B, videos S2V and S3V.

**Figure 2.**
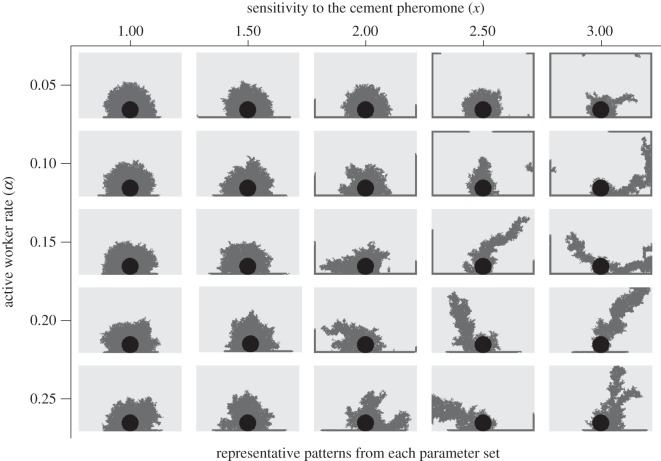
Representative patterns generated by changing individual workers’ parameters in construction simulations with 400 workers. Patterns created by workers highly sensitive to the cement pheromone are on the right and those created by less-sensitive workers are on the left. Patterns created by a small number of active workers are along the top, and patterns created by a large number of active workers are along the bottom.

**Figure 3.**
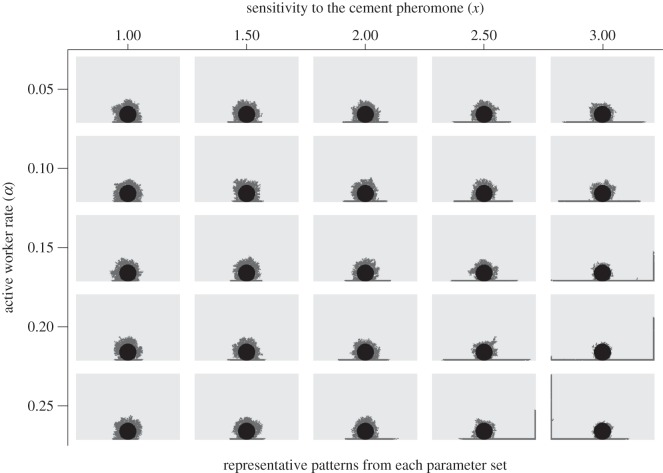
Representative patterns generated by changing individual workers’ parameters in construction simulations with 50 workers.

**Figure 4.**
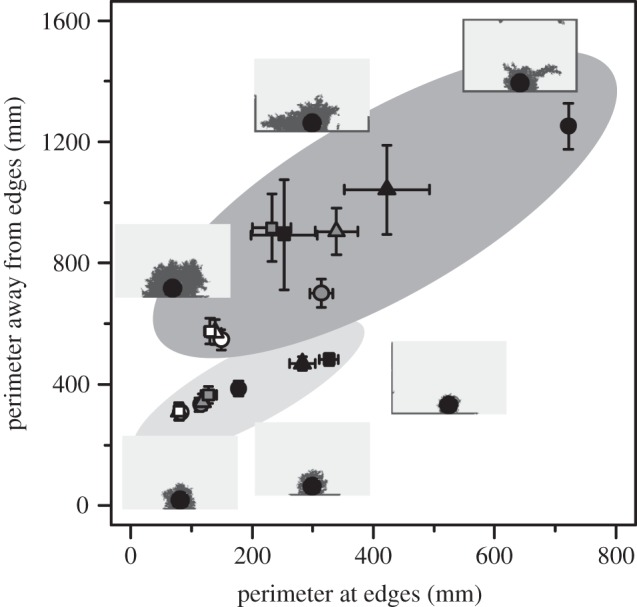
The perimeters of patterns created by simulations. The relationship between coloured area and the number of individuals is the same as figure 1. Different shapes represent differences in rate of active workers (circles, *α*=0.05; triangles, *α*=0.15; squares, *α*=0.25). Different colours represent differences in sensitivity to the cement pheromone (white, *x*=1; grey, *x*=2; black, *x*=3). Bars represent means±s.d. (*n*=100). To show ranges of structures created by each parameter set, s.d. is used instead of s.e.

Our simulations generated various patterns similar to those of actual termite structures (figures 2 and 3). When we changed two individual worker characteristics; the rate of active workers and sensitivity to the cement pheromone, the generated patterns varied dramatically. In 400 agents groups, shelter tube patterns were created by fewer, highly sensitive workers (especially when *α*=0.05, *x*=3.0; figure 2), mat patterns by less-sensitive workers at all active worker rate (*x*=1.0; figure 2) and intermediate patterns by intermediate worker characteristics (*α*=0.25, *x*=2.0; figure 2). In 50 agents groups, the length of shelter tubes became longer according to the increase of the sensitivity and the number of agents engaging in construction (figure 3). On the other hand, with low sensitivity, whatever the number of active workers was, agents could not construct long shelter tubes. For building shelter tube patterns, 50 agents groups also needed high sensitivity to the pheromone (figure 3). By changing parameters of individuals among groups, our simulations could recreate very similar patterns to the observed patterns in our empirical experiments (figures 1 and 4).

**Figure 5.**
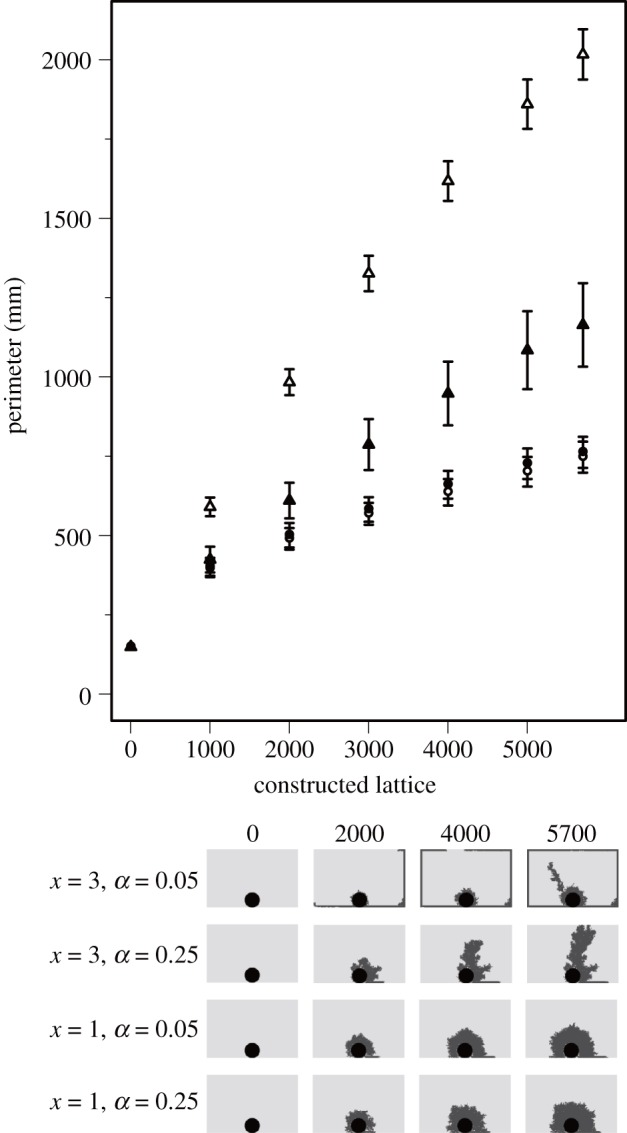
The building process of patterns generated in construction simulations with 400 workers. Different shapes represent differences in sensitivity to the cement pheromone (circle, *x*=1; triangle, *x*=3). Different colours represent differences in the rates of active workers (white, *α*=0.05; black, *α*=0.25). Bars represent means±s.d.

The patterns varied with the amplification strength of construction behaviour which was caused by different degrees of positive feedback. The construction process in simulations of 400 agents groups was shown in figure 5. When the sensitivity to the cement pheromone was low (small value of *x*), a low degree of positive feedback caused simultaneous building at many candidate locations. This led to the circular growth of nest construction with the increase ratio of perimeters being very low (circle points in figure 5). By contrast, when sensitivity to the cement pheromone was high (large *x*), a high degree of positive feedback caused localized construction. This process resulted in creating shelter tube patterns, which increases perimeters at high slope (open triangle points in figure 5). This phenomenon was prominent in the simulations with a small number of active workers. In the simulations with a large number of active workers, local crowding sent agents to other candidate locations within one time step, which resulted in simultaneous construction at many candidate locations. This interfered with the high ratio increase of perimeters (closed triangle points in figure 5).

## 3. Discussion

In our laboratory experiment, termites showed clear intercolonial variation in shelter tube construction, even under identical environments and group sizes. Our two-dimensional lattice model successfully demonstrated that this variation can be reproduced by changing the parameters of individual workers even in the same building algorithm, which suggests that termite colonies constructing different structures possess varying physiological parameters in building behaviour. In addition, our sensitivity analysis demonstrated that the simulation results are robust to a range of values of parameters (electronic supplementary material, appendix A). These results provide a novel possible mechanism of creating intraspecific variation in structures which can be tested in various social organisms in further studies.

The amplification process through positive feedback is an essential component in various self-organized collective behaviours of social insects such as aggregation, foraging, defence, clustering and structure building [18,23–26]. For example, in ants, foraging intensity on two different trails has been theoretically suggested to become increasingly asymmetric as the degree of positive feedback for recruitment becomes high [27,28]. A similar scenario can be applied to shelter tube construction in termites. Our model shows that variation among colonies can be created by differences in positive feedback, which are regulated by sensitivity to the cement pheromone. Colonies with individuals with high sensitivity are strongly attracted to the cement pheromone, causing a high degree of positive feedback and construction to be limited to fewer locations. By contrast, individuals with low sensitivity are only weakly attracted to the cement pheromone, causing construction to occur in multiple candidate locations and resulting in circular structures, i.e. mats.

How colonies of social insects vary in behaviour is a crucial question [29]. In social insect colonies, workers perform a variety of tasks including foraging, brood care and nest construction. As the needs of the colony change, colonies adjust the number of workers engaged in each task [30]. The needs of shelter tube construction can vary among colonies depending on demographic stage [31,32], nutritional conditions [33–35] and abundance of dispersal individuals [36]. The colony variation of shelter tube patterns may reflect the need of construction of their original colonies. Examining how colony conditions affect shelter tube construction will provide a stepping stone to understand the adaptive significance of the intercolonial variation.

Understanding intercolonial variation in structures is a key to understanding the evolution of collective building in social insects. Although natural selection operates on the structures themselves, colony-level differences stem from individual-level responses. Considering the complexity of structures, we may suppose fundamental change of behavioural algorithms to create large variations in nest construction. In this study, however, we showed that a wide variety of termite construction can be reproduced by simply changing two individual-level characteristics of workers. This may also explain various self-organized behaviours performed by many group-living organisms. By linking changes in individuals to variation in group-level patterns, our study provides important insight for clarifying the evolution of self-organization systems that create complex and sophisticated patterns from simple behavioural rules.

## Supplementary Material

ESM Table and Figures: Complementary methods for empirical experiments and simulations.

## Supplementary Material

Appendix A: Sensitivity Analysis of two dimensional lattice model Appendix B: The code of the simulations. Appendix C: The whole data of empirical experiments and simulations.

## Supplementary Material

STsimulation15_10_01.txt

## Supplementary Material

150623_data.xlsx

## Data accessibility

Data from empirical experiments and simulations are available as the electronic supplementary material.
