# Supplementary material for: Emergence of intercolonial variation in termite shelter tube patterns and prediction of its underlying mechanism
Source: R Soc Open Sci. 2015 Nov 4;2(11):150360. doi: 10.1098/rsos.150360 (PMC4680612; doi:10.1098/rsos.150360)
Supplement: ESM Table and Figures: Complementary methods for empirical experiments and simulations. [file rsos150360supp1.pdf]

Electronic Supplementary Material (ESM)

Table S1

Figure S1 - S6

Emergence of intercolonial variation in termite shelter tube patterns and prediction of its underlying mechanism

Nobuaki Mizumoto*, Kazuya Kobayashi & Kenji Matsuura

Laboratory of Insect Ecology, Graduate School of Agriculture, Kyoto University, Kyoto 606-8502, Japan.

Phone & Fax: +81-75-753-6136

* Correspondence: N. Mizumoto, Laboratory of Insect Ecology, Graduate School of Agriculture, Kyoto University, Kyoto 606-8502, Japan.

E-mail address: mizumoto.nobuaki.75a@st.kyoto-u.ac.jp (N. Mizumoto).

Table S1

The survival rate of each group of workers on 30 day. The values of groups whose survival rate ≤ 0.5 are written in red, which were excluded from further analysis.

Group type	replicates 1	replicates 2	replicates 3	replicates 4	replicates 5
400 workers					
colony A	0.7600	0.3975	0.8375	0.7350	0.3750
colony B	0.7325	0.7150	0.7575	0.6800	0.6950
colony C	0.6325	0.5175	0.5675	0.7100	0.4450
colony D	0.5675	0.6625	0.5600	0.5750	0.5950
colony E	0.4850	0.6550	0.6375	0.6975	0.6775
50 workers					
colony A	0.84	0.90	0.80	0.86	0.50
colony B	0.80	0.92	0.68	0.82	0.78
colony C	0.78	0.74	0.76	0.88	0.80
colony D	0.76	0.74	0.64	0.80	0.74
colony E	0.82	0.92	0.74	0.66	0.90

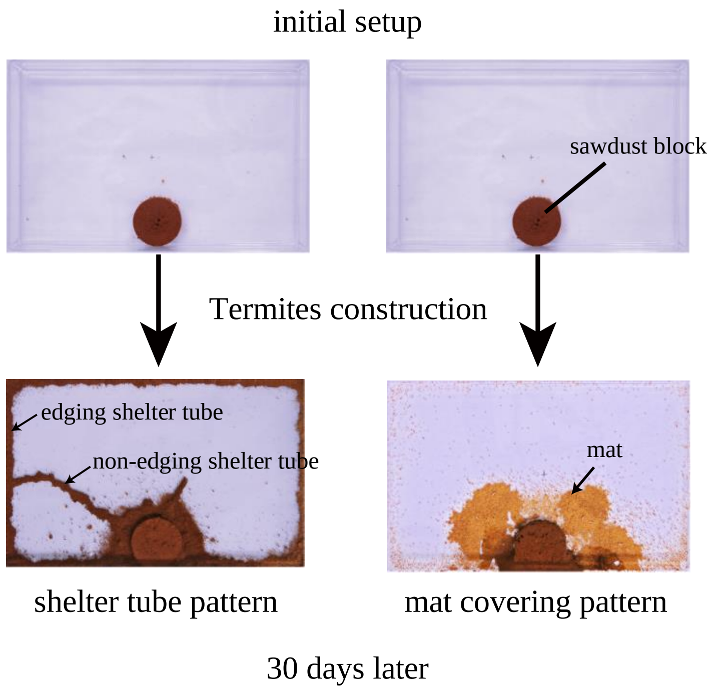

Figure S1: Initial experimental setup and structures constructed by 400 workers of termite 30 days later. Two types of shelter tubes and mat are indicated by *arrows* in the photographs. We created 50 initial setup for experiments (5 colonies × 2 group size × 5 replicates).

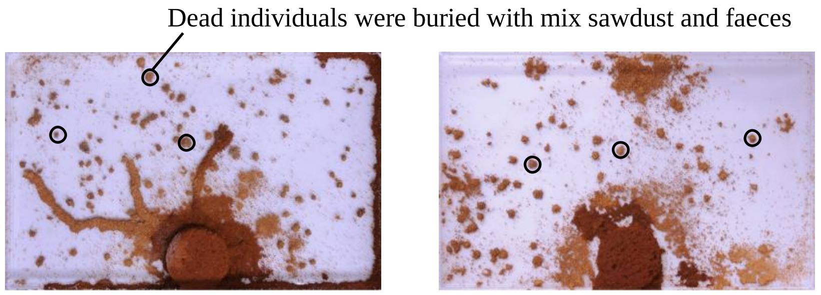

Figure S2: The photographs of groups whose survival rates were low.

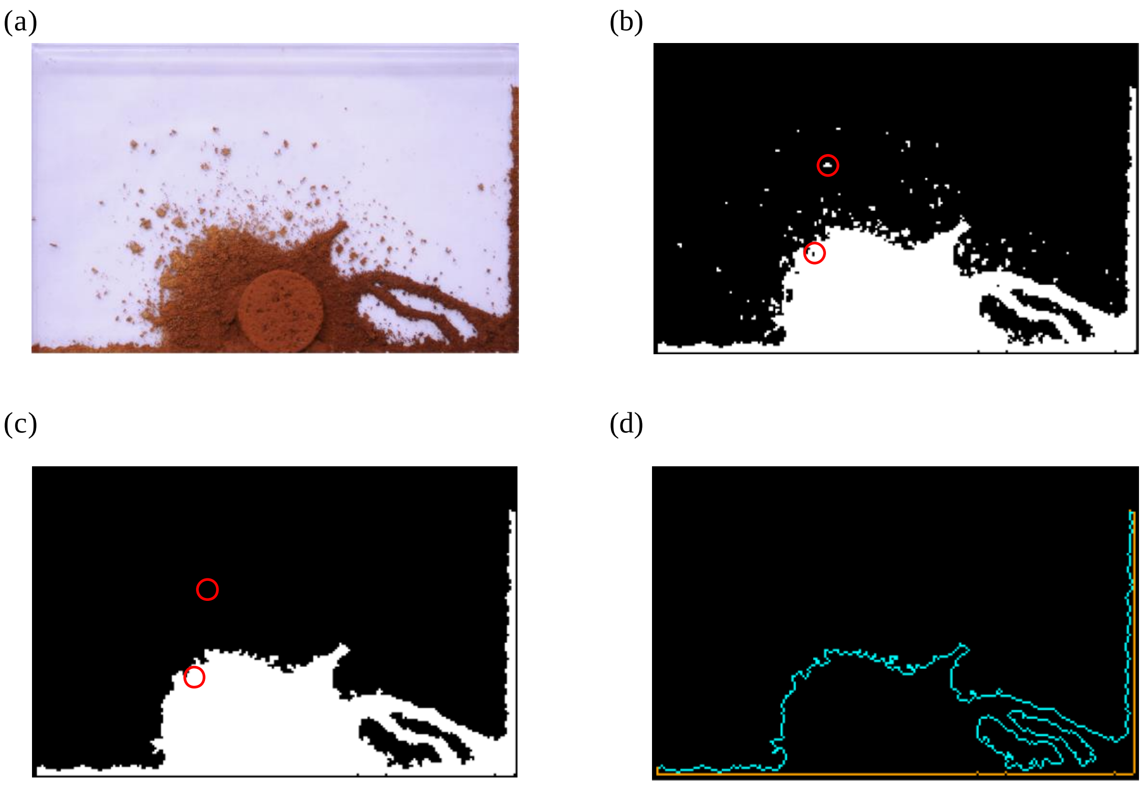

Figure S3: The method of conversion of photographs. (a) The photograph before the conversion. (b) The photographs are adjusted so that 1 pixel represented ~1 mm (220*140 pixels) and are converted to black-and-white so brown areas (nest construction) became white and backgrounds became black. (b-c) To measure the perimeter by border between black and white, we eliminated small pieces (white pieces < 50 pixels, black pieces < 10 pixels, some of them are circled in red), that arises from sawdust, termite faeces or termite individuals. (d) Then, the perimeters were separately measured along edges (3 mm from edge of the container) and away from edges, where former is lined in orange and later in blue.

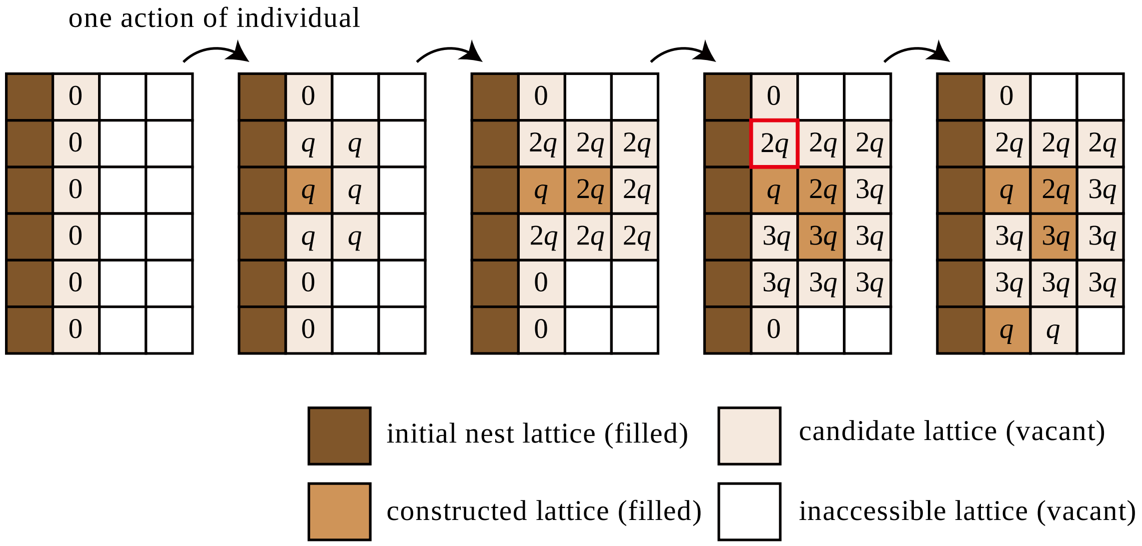

Figure S4: The rule of pheromone accumulation. Construction continues from left to right by filling vacant lattices which are adjacent to the existing structure. Number and letters in lattices are the quantities of pheromone in each lattice. The quantities of cement pheromone for candidate lattices are defined as the largest value of the constructed lattices neighbouring to each of the candidate lattices. For example, the quantity of pheromone for the lattice i (the red squared lattice) is defined as $2q$ which is the largest value of the constructed lattices neighbouring to the lattice i.

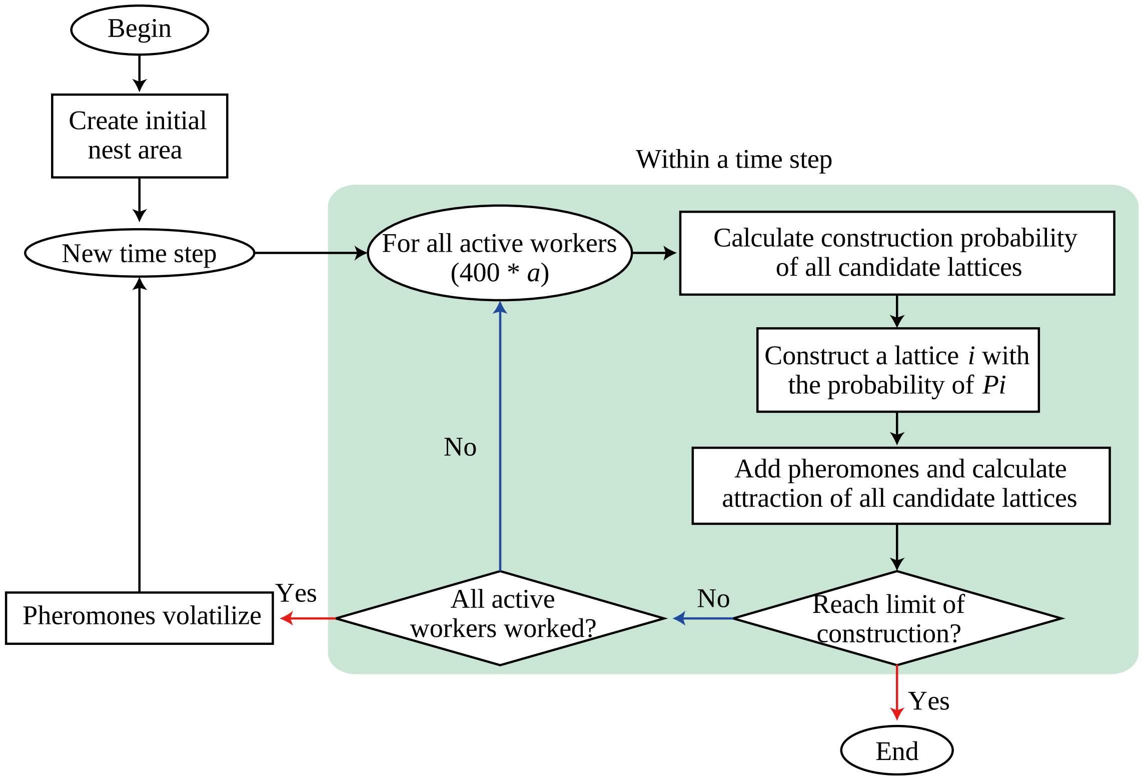

Figure S5: Flowchart of the construction simulation.

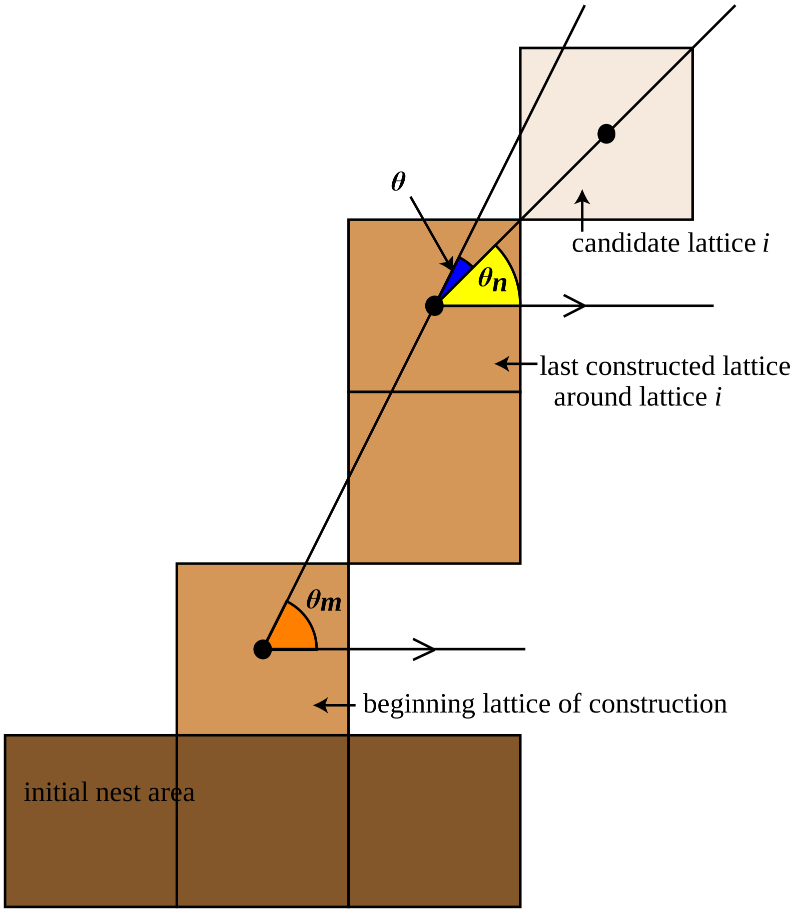

Figure S6: The parameter of constructing direction *vi*. *vi* is determined by θ which is the angle between the direction to the last constructed lattice from the lattice where construction begins (θm) and the direction to the candidate lattice *i* from the last constructed lattice around the candidate lattice (θn). θ is computed by θm - θn. If the candidate lattice *i* is in the opposite direction toward the beginning lattice from the last constructed neighbour lattice ($\theta n = \theta m$), *vi* is 1. If the lattice i is in the direction towards the beginning lattice ($\theta n = \pi + \theta m$), *vi* is 0.
