# Supplementary material for: Emergence of intercolonial variation in termite shelter tube patterns and prediction of its underlying mechanism
Source: R Soc Open Sci. 2015 Nov 4;2(11):150360. doi: 10.1098/rsos.150360 (PMC4680612; doi:10.1098/rsos.150360)
Supplement: Appendix A: Sensitivity Analysis of two dimensional lattice model Appendix B: The code of the simulations. Appendix C: The whole data of empirical experiments and simulations. [file rsos150360supp2.pdf]

Electronic Supplementary Material (ESM)

Appendix A: Sensitivity Analysis of two dimensional lattice model

Emergence of intercolonial variation in termite shelter tube patterns and prediction of its underlying mechanism

Nobuaki Mizumoto*, Kazuya Kobayashi & Kenji Matsuura

Laboratory of Insect Ecology, Graduate School of Agriculture, Kyoto University, Kyoto 606-8502, Japan.

Phone & Fax: +81-75-753-6136

* Correspondence: N. Mizumoto, Laboratory of Insect Ecology, Graduate School of Agriculture, Kyoto University, Kyoto 606-8502, Japan.

E-mail address: mizumoto.nobuaki.75a@st.kyoto-u.ac.jp (N. Mizumoto).

Sensitivity Analysis

In this Electronic Supplementary Material, we investigated the sensitivity of the model results to the quantity of cement pheromone added by an agent (*q*), the evaporation rate of the cement pheromone (*r*), the lattice preference irrespective of pheromone quantity around edges of the container (*ki* around edges: k_{edge}) and the another rule for construction direction. In simulations for *q* and *r*, we changed these two values simultaneously. In simulations for k_{edge}, only k_{edge} was changed. We used 400 agents and parameters (α, *x*) = (0.05, 3), (0.15, 2) and (0.25, 1) which showed representative patterns of shelter tubes, intermediate and mat covering in main result (figure 2). For each parameter set, we ran 100 simulations. Simulation results were analysed in the same way as the empirical experiment.

Quantity of cement pheromone and Evaporation rate

We tested the effect of these parameters (*q* and *r*) on the simulation results using two sets of parameters. One was a set of narrow range which was composed of 5 values of *q* and *r* ranging from 0.1 to 0.9 at an interval of 0.2. The other was a set of wide range which was composed of 5 values of *q* increasing from 0.003 to 30.0 by ten times and 5 values of *r* increasing from 0.00007 to 0.7 by ten times. In total, 49 parameter sets were examined for the combination of *q* and *r*.

We could not re-create the observed patterns by only changing parameters *q* and *r* in narrow range, using 3 types of agents (compare figure A1a with figure A1b-d). With parameters (α, *x*) = (0.15, 2) and (0.25, 1), the effect of *q* and *r* on resulting patterns was relatively slight (figure A1c,d, figure A2). On the other hand, with parameters (α, *x*) = (0.05, 3), patterns dramatically varied with the change of *q* and *r* (figure A1b, figure A2). There were some sets of *q* and *r* re-creating observed patterns other than (*q*, *r*) = (0.3,

0.7), such as (q, r) = (0.1, 0.9), (0.5, 0.7), (0.5, 0.5) and (0.7, 0.5).

When we changed parameters q and r in wide range, the patterns also dramatically varied with change of r especially in high value of q (figure.A3 and figure. A4). In this study, however, as we conducted experiments in the same environmental conditions, it should be difficult to assume that different colonies had extremely different evaporation rate. This variation may explain interspecific variation rather than intraspecific variation, where different species should use different chemical for cement pheromone [1].

Preference irrespective of pheromone quantity

We tested the effect of the lattice preference irrespective of pheromone quantity at edge zone (k_{edge}) using 5 values ranging from 1 to 3 at an interval of 0.5. According to the increase of k_{edge}, perimeters along edges and away from edges also increased. With the value of $k_{edge} \geq 2.0$, patterns similar to the observed patterns in empirical experiment were re-created by changing α and x (figure A5, A6).

Another rule for the effect of direction of construction

In our simulations, we included the effect of construction direction on the attraction of lattices by *vi* because shelter tubes are constructed in a direction away from the nest. The direction of shelter tube construction can be described by θ which is the angle between the direction to the last-constructed lattice from the first-constructed lattice and the direction to the candidate lattice from the last-constructed lattice (see electronic supplementary material, figure S6). In the main manuscript, we attached 0-1 to θ by using cosine curve, where the relationship between θ and *vi* was nonlinear. However there are some other rules which can output 0-1 responding to directions. Therefore, here we tested another rule for deciding directions.

As another rule for deciding direction, we directly attached θ to 0-1. Here the parameter of construction direction *vi* is set as:

$$v_i = 1 - \frac{\theta}{\pi},$$

where the relationship between θ and vi is linear. Using this rule, we conducted simulations, where values of α was ranging from 0.05 to 0.25 at an interval of 0.05 and the values of *x* was ranging from 1 to 3 at an interval of 0.5.

Simulations with another rule for deciding direction of construction generated similar patterns to those of main manuscript (figure A7, A8). Like the simulations using cosine curve, shelter-tube patterns were created by fewer, highly sensitive workers, mat patterns by less sensitive workers at all active worker rate and intermediate patterns by intermediate worker characteristics (figure A8).

Reference

1 Khuong, A., Theraulaz, G. & Jost, C. 2011 A computational model of ant nest morphogenesis. *In Proceedings of the Eleventh European Conference on the Synthesis and Simulation of Living Systems, Advances in Artificial Life, ECAL2011.* , 404–411.

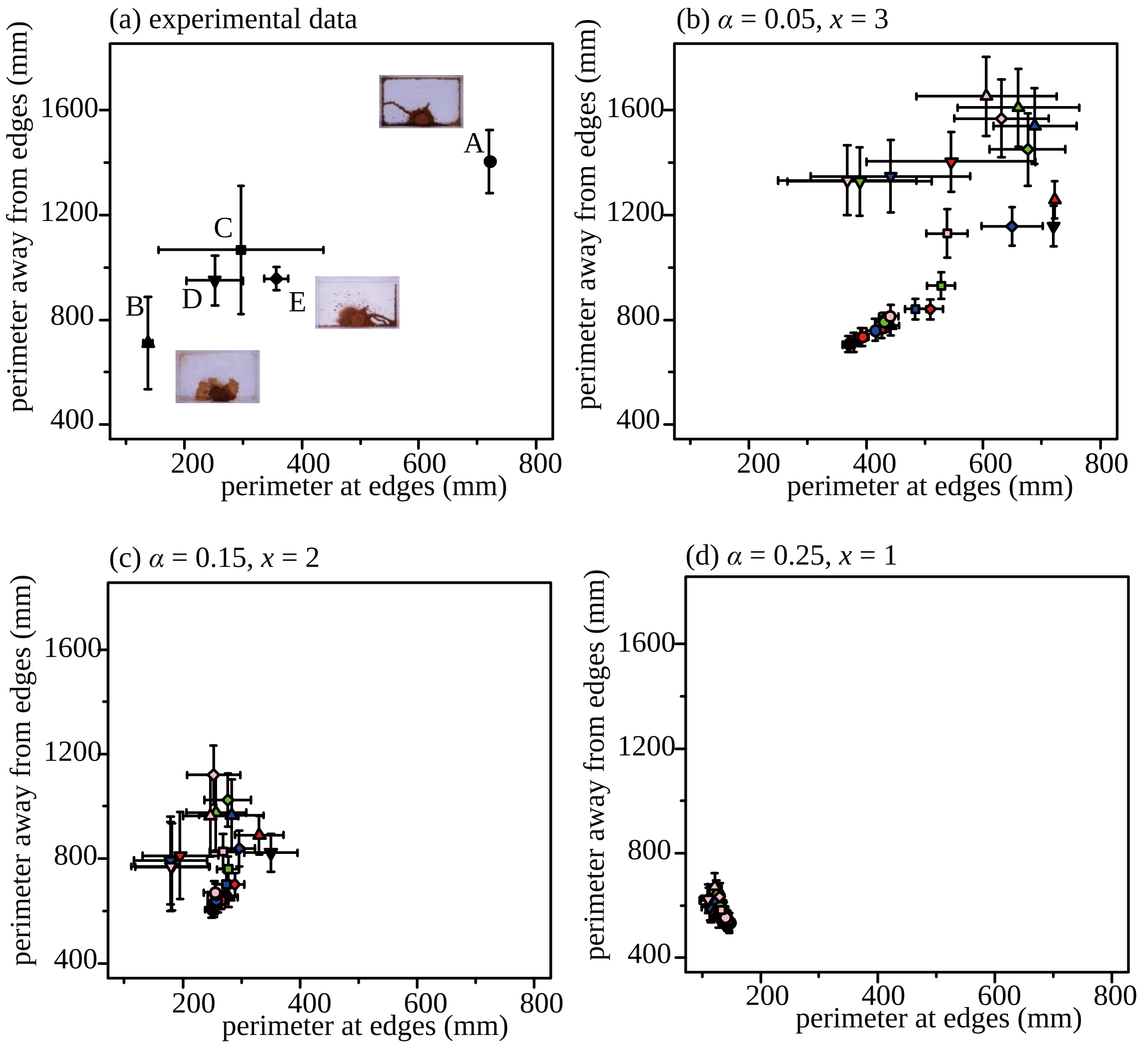

Figure A1. Effect of q and r in narrow range on the simulation results. (a) Observed colony variation in termite shelter-tube construction. Bars represent means± S.E. (b-d) The effect of the quantity of cement pheromone added by an agent (q) and the evaporation rate of the cement pheromone (r) on the structures. Different colours represent differences in q (black, q = 0.1; red, q = 0.3; blue, q = 0.5; green, q = 0.7; pink, q = 0.9). Different shapes represent differences in r (circle, r = 0.1; square, r = 0.3; diamond, r = 0.5; triangle, r = 0.7; reversed triangle, r = 0.9). Bars represent means± S.D. (n = 100).

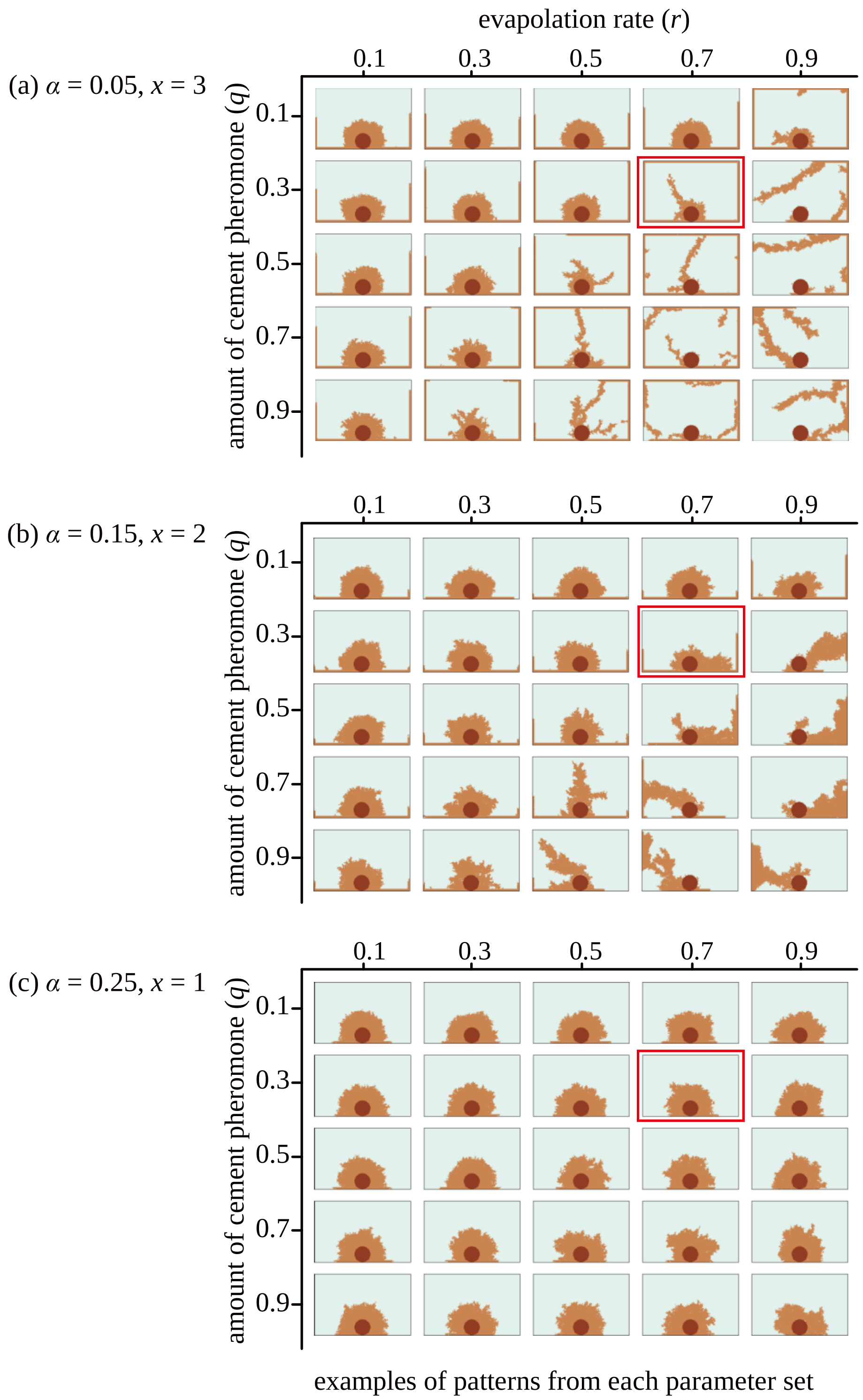

Figure A2. Representative patterns generated by changing individual characteristics in construction simulations. q and r ranging from 0.1 to 0.9 at an interval of 0.2. The patterns created by the same parameter set with main simulation are boxed in red.

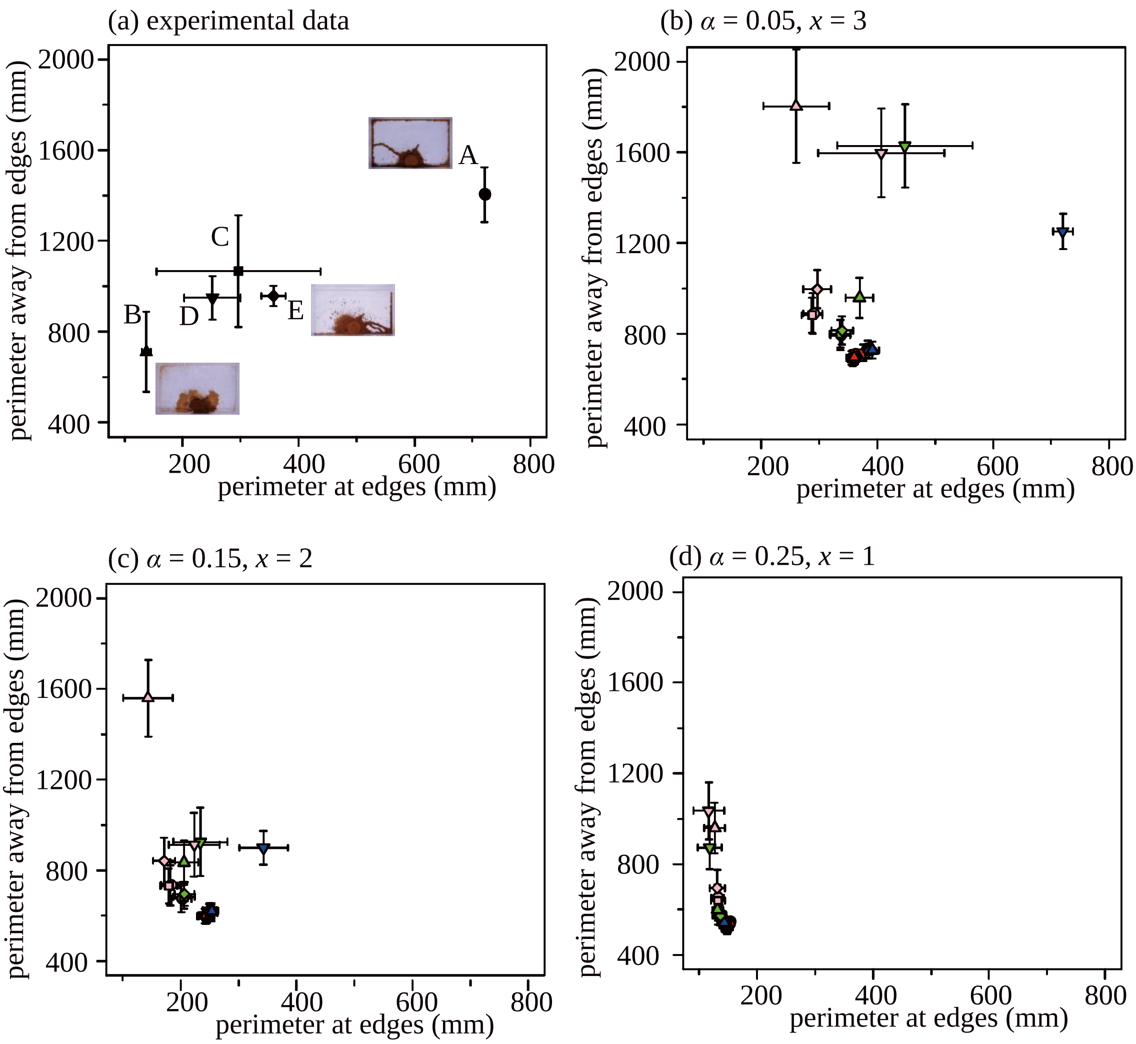

Figure A3. Effect of q and r in wide range on the simulation results. (a) Observed colony variation in termite shelter-tube construction. Bars represent means± S.E. (b-d) The effect of the quantity of cement pheromone added by an agent (q) and the evaporation rate of the cement pheromone (r) on the structures. Different colours represent differences in q (black, q = 0.003; red, q = 0.03; blue, q = 0.3; green, q = 3; pink, q = 30). Different shapes represent differences in r (circle, r = 0.00007; square, r = 0.0007; diamond, r = 0.007; triangle, r = 0.07; reversed triangle, r = 0.7). Bars represent means± S.D. (n = 100).

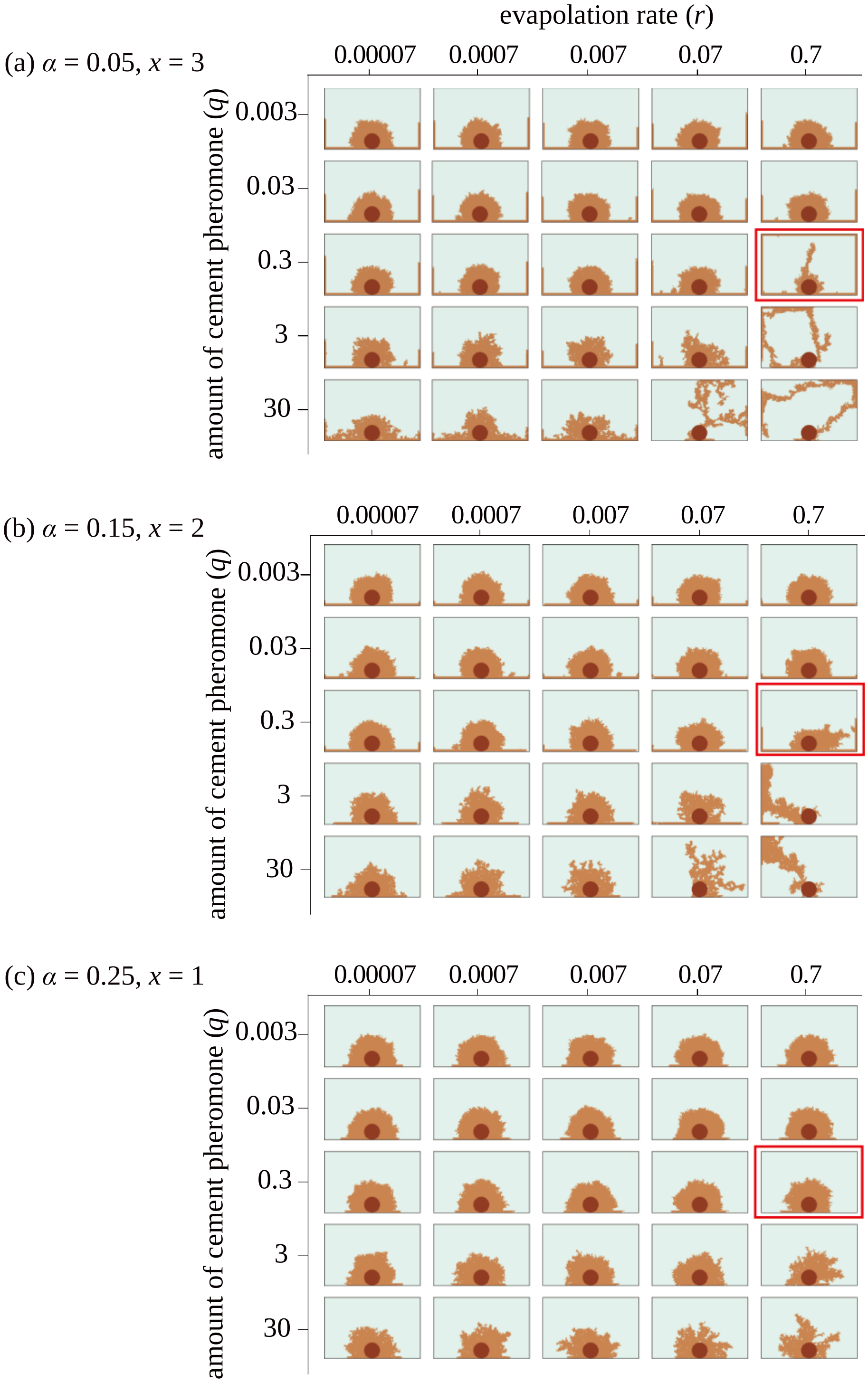

Figure A4. Representative patterns generated by changing individual worker's characteristics in construction simulations. q increases from 0.003 to 30.0 by ten and r increases from 0.00007 to 0.7 by ten. The patterns created by the same parameter set with main simulation are boxed in red.

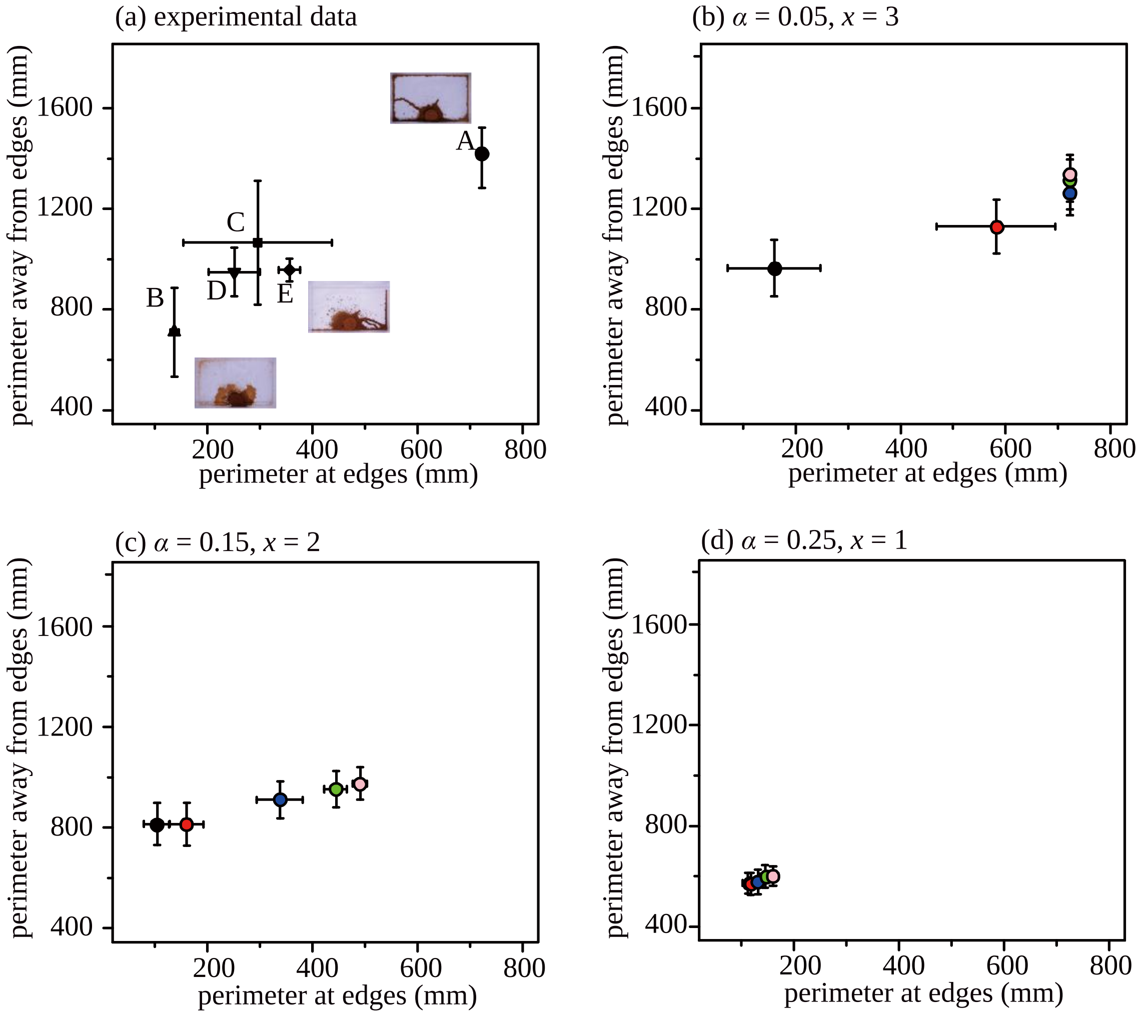

Figure A5. The effect of the lattice preference irrespective of pheromone quantity around the edges of containers (k_{edge}) on the simulation results. (a) Observed colony variation in termite shelter-tube construction. Bars represent means± S.E. (b-d) The effect of k_{edge} on the structures. Different colours represent differences in k_{edge} (black, k_{edge} = 1.0; red, k_{edge} = 1.5; blue, k_{edge} = 2.0; green, k_{edge} = 2.5; pink, k_{edge} = 3.0). Bars represent means± S.D. (n = 100).

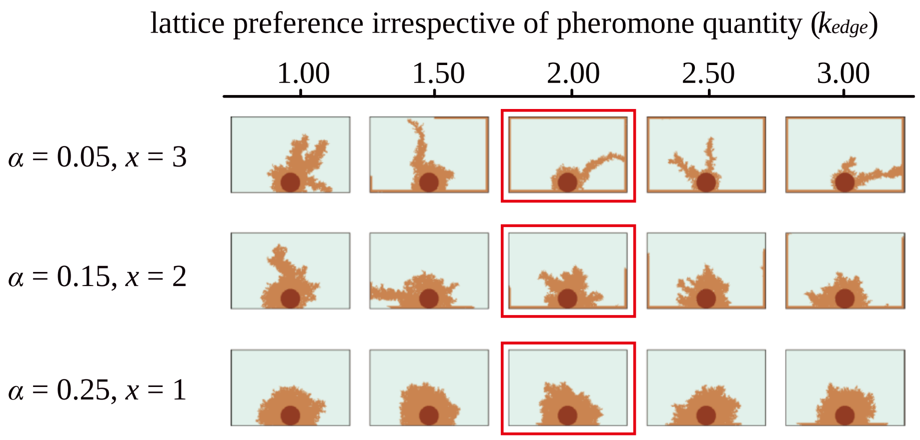

Figure A6. Representative patterns generated by changing the lattice preference irrespective of pheromone quantity around the edges of containers (k_{edge}). The patterns created by the same parameter set with main simulation are boxed in red.

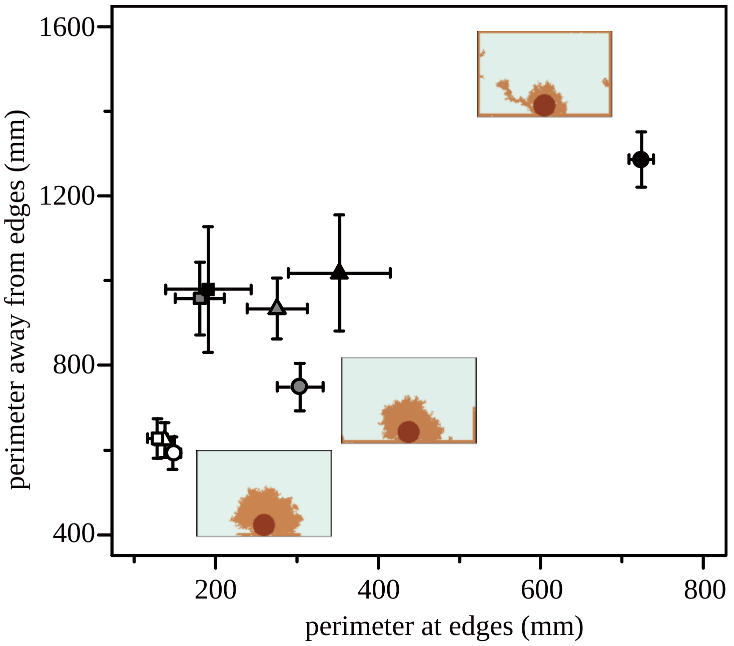

Figure A7. Patterns created by simulations using another rule of construction direction. Different shapes represent differences in sensitivity to the cement pheromone (circle, x = 1; triangle, x = 2; square, x = 3). Different colours represent differences in the rate of active workers (white, α = 0.05; grey, α = 0.15; black, α = 0.25). Bars represent means ± S.D.(n = 100).

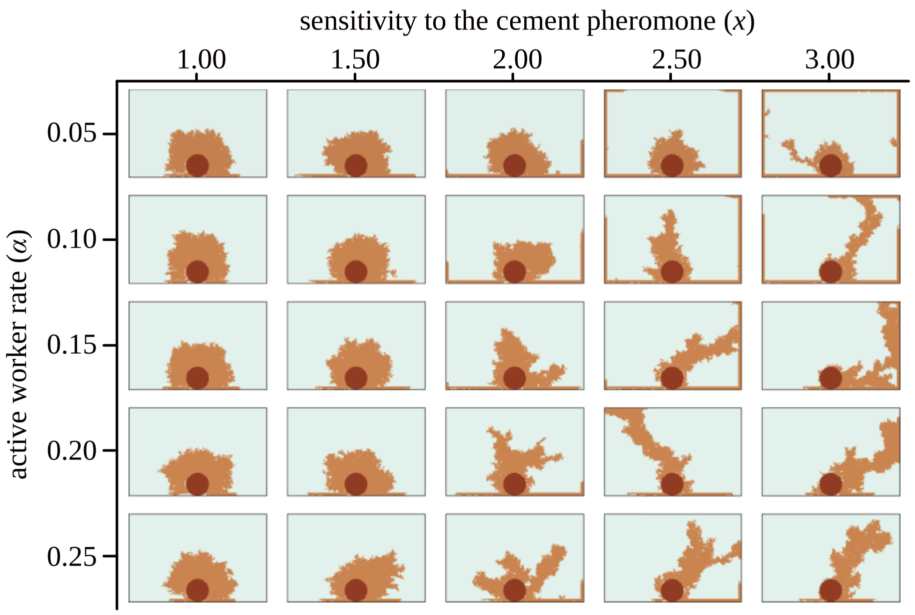

Figure A8. Representative patterns generated by changing individual worker characteristics in construction simulations with another rule of construction direction.
